# New Hybrid Ethylenediurea (EDU) Derivatives and Their Phytoactivity

**DOI:** 10.3390/ijms25063335

**Published:** 2024-03-15

**Authors:** Maxim S. Oshchepkov, Leonid V. Kovalenko, Antonida V. Kalistratova, Sergey V. Tkachenko, Olga N. Gorunova, Nataliya A. Bystrova, Konstantin A. Kochetkov

**Affiliations:** 1 Department of Chemistry and Technology of Biomedical Drugs, Mendeleev University of Chemical Technology of Russia, Miusskaya Sq. 9, 125047 Moscow, Russiakalistratova.a.v@muctr.ru (A.V.K.);; 2 A. N. Nesmeyanov Institute of Organoelement Compounds, Russian Academy of Sciences, 28 Vavilova St., 119991 Moscow, Russia; olg111@yandex.ru (O.N.G.); bystrova17.11@inbox.ru (N.A.B.)

**Keywords:** phytohormones, cytokinins, phenylureas, ethylenediurea derivatives, wheat seeds, germination, relative water content

## Abstract

Natural and synthetic phytohormones are widely used in agriculture. The synthetic cytokinin ethylenediurea (EDU) induces protection in plants against ozone phytotoxicity. In our study, new hybrid derivatives of EDU were synthesized and tested for phytoactivity. The germination potential (Gp), germination of seeds (G), and relative water content in leaves (RWC), characterizing the drought resistance of plants, were determined. The results of laboratory studies showed that EDU and its hybrid derivatives have a positive effect on root length, the growth and development of shoots, as well as the ability of plants to tolerate stress caused by a lack of water.

## 1. Introduction

**Figure 1:**
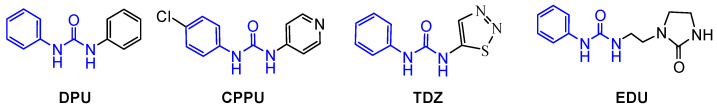
Known cytokinins: *N,N*′-diphenylurea (DPU), *N*-phenyl-*N*′-(2-chloro-4-pyridyl)urea (CPPU), *N*-phenyl-*N*′-1,2,3-thiadiazol-5-ylurea (TDZ), *N*-[2-(2-oxo-l-imidazolidinyl)ethyl]-*N*′-phenylurea (EDU).

Global climate change has a negative impact on global crop production, threatening food safety around the world [1]. Exogenous phytohormones such as auxins (Auks) and cytokinins (Ctks) have been used recently to improve seed germination, stress, and disease resistance and increase crop yields [2]. According to the marketing research company Future Market Insights [3], the production and demand for exogenous cytokinins will double in the next decade. They stimulate the mobilization and transport of nutrients and changes in all growth processes. The treatment of crops with exogenous phytohormones can be used to prevent the lodging of cereals [4], synchronize fruit ripening, delay aging, and improve qualitative and quantitative yield indicators [5]. Today, one of the key types of exogenous cytokinins is *N,N*′-diphenylurea (DPU) and its analogues: *N*-phenyl-*N*′-(2-chloro-4-pyridyl)urea (forchlorfenuron, CPPU), *N*-phenyl-*N*′-1,2,3-thiadiazol-5-ylurea (TDZ), *N*-[2-(2-oxo-l-imidazolidinyl)ethyl]-*N*′-phenylurea (EDU), etc. [6] (Figure 1).

Ethylenediurea (EDU) occupies a special place in the range of urea derivatives. It consists of two moieties: an imidazolidinone and an N-phenylurea residue, connected by a short linker. EDU can be used to protect plants from tropospheric ozone (O_3_), whose content in the air has increased significantly over the past thirty years [7]. The increased concentration of tropospheric ozone reduces crop yields due to oxidative stress. EDU mitigates the harmful effects of oxidative stress, improves photosynthesis and growth, and increases biomass and productivity. The use of EDU helps in obtaining good harvests of maize [8], rice [9,10], and peanuts [11], increases the growth and biomass of peas [12] and legumes [13,14], and improves the growth, nutritional properties, and yield of wheat grain [15,16,17,18]. It also protects seedlings of Japanese larch [19,20] and willow [21], which are important plants for cultivation as sources of bioenergy.

It was found in [22] that the rate of application of EDU required to protect plants from ozone depends on the features of the crop and environment and typically ranges from 0.02 to 3.0 kg per hectare. EDU can be applied to soil [23] or used as a foliar spray [21]. The optimal concentration for foliar spraying is 500 ppm of EDU, which provides good protection for flowers, herbaceous plants, and some types of tree seedlings. 

In addition, EDU has cytokine-like activity at a concentration of 2 × 10^−5^ M [24]. In terms of its biological properties, this compound is close to natural adenine-type Ckts, such as kinetin (KIN) [5]. It binds to the same site of cytokinin receptors as natural derivatives of N^6^-substituted adenine, leading to the activation of a two-component signaling system [25].

The main idea of our work was to obtain a series of hybrid derivatives of EDU (**I**–**IX**) and to study their effect on the growth and development of wheat seeds (*Triticum aestivum* L.) in comparison with the known compounds kinetin (KIN) and *N*-2-hydroxyethyl-2-oxo-imidazolidine (IM).

## 2. Results and Discussion

### 2.1. Chemical Synthesis

**Figure 2:**
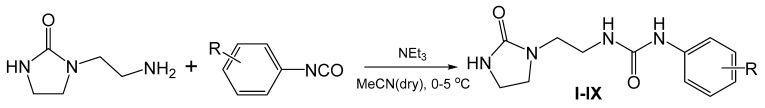
Preparation of EDU derivatives (**I**–**IX**).

New EDU derivatives (**I**–**IX**) were obtained using known methods (according to Figure 2) from 2-imidazolidinone with an aminoethyl substituent by interaction with various aryl isocyanates in the presence of triethylamine in anhydrous acetonitrile, as described in [26,27,28]. The main physicochemical characteristics of compounds (**I**–**IX**) are given in the Supplementary Materials.

### 2.2. Laboratory Tests

We have previously shown that EDU derivatives affect the growth and development of seeds, and the optimal concentration for use is 4·10^−5^ M [3]. In this study, wheat seeds were treated with compounds **I**–**IX** according to the described method (S.II), by which the following indicators were determined: germination potential (Gp, %), germination (G, %), root length (root, cm), shoot height (shoot, cm), and relative content water (RWC, %). The results of the experiments are presented in Table 1.

It is known that the levels of cytokinins in plants are regulated by the enzyme cytokinin oxidase/dehydrogenase [5]. However, the mechanism of action of ethylenediurea derivatives is not fully understood. In plants, the phenyl moiety of ethylenediurea compounds binds to the same hydrophobic pocket as the isoprenoid tail of trans-zeatin (tZ). The urea moiety establishes polar interactions in the site of action that are very similar to those observed for *N*^7^ and *N*^6^ in adenine-type complexes [29]. Therefore, urea derivatives **I**–**IX** are probably also able to bind to the cytokinin binding site in the receptor, which possibly determines their cytokinin activity.

**Figure 3:**
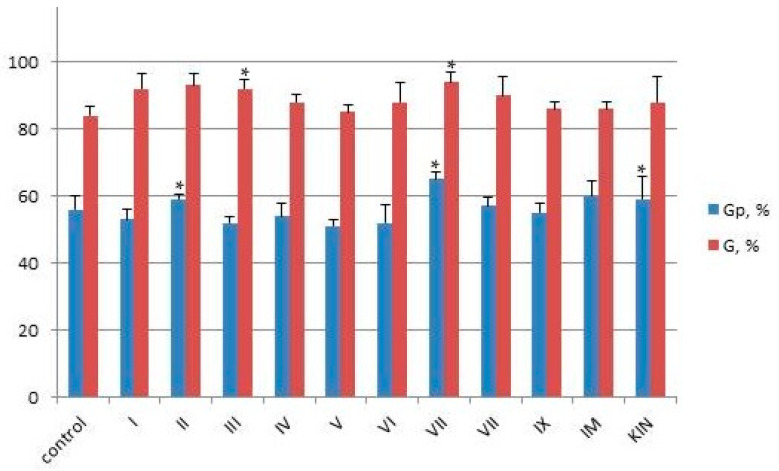
Germination potential (Gp, %) and germination (G, %) of wheat seeds treated by compounds **I**–**IX**, **IM**, **KIM**. Control was treated only by distilled water. Each treatment group contained 4 replicates (600 seeds in each replicate). Online calculator was used to analyze the data, which are presented as mean ± standard deviation. Statistical significance was determined by Student’s *t* test based on *p* < 0.01 (*).

The life cycle of a plant begins with the germination stage. To simulate the development of seeds, the first day of the experiment was carried out in the dark. The appearance of visible roots after 24 h was used as a morphological marker of germination potential (Gp) and calculated using Formula (1). The results are shown in Figure 3.

The germination (G) of the seeds was calculated on the seventh day by Formula (2), according to the recommendation of the International Seed Testing Association (ISTA) [30]. It was found that seeds treated with compound **VII** had higher values of Gp (65%) and G (94%) compared to the control (56% and 84%) and those treated by **IM** (60% and 86) and KIN (59% and 88%). According to this, it was superior among the tested compounds. The germination potential and germination of seeds treated by **I**, **II,** and **III** also had high values (average Gp value—above 50%; average G value—above 90%). This may be due to the presence of a chlorine atom in the aromatic ring as an electronegative and hydrophobic substituent, which can also lead to increased binding of the urea moiety to cytokinin receptors responsible for the activation of the two-component signaling system [25]. The germination potential and germination of seeds treated by compounds **IV**–**VI** had an average Gp value of above 50% and an average G value of above 85%. These are also statistically higher than the average control values, which indicates good opportunities for a further search for leading compounds among this class of cytokinin-like synthetic compounds. It should be noted that, in these experiments, shoots appeared on the second day, which is significantly earlier than the results we obtained in other investigations [3,31].

Drought stress is the most harmful environmental factor, and it is described as a multifactorial environmental stress because it causes extensive changes in plant morphology, physiology, and biochemistry [32]. The advanced architecture of root systems helps to improve the adaptation of plants to changes in the environment and is one of the key characteristics that determine the yield in normal, stressful, and (primarily) drought conditions [3,31,33].

**Figure 4:**
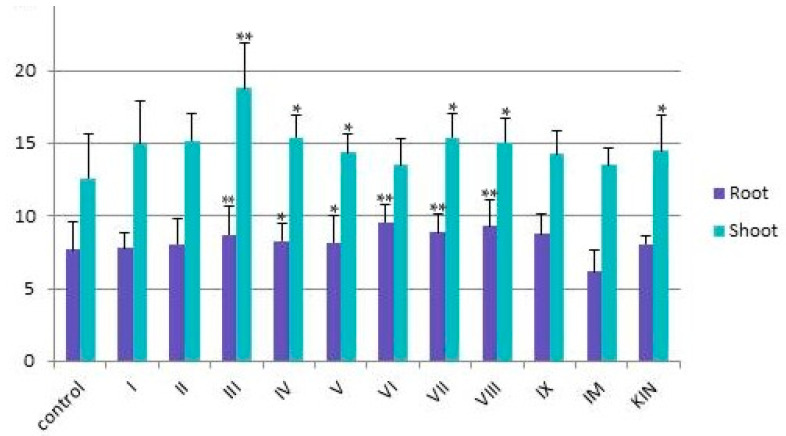
Root length (cm) and shoot height (cm) of wheat seeds treated by compounds **I**–**IX**, **IM**, and **KIM** and controls. Online calculator was used to analyze the data, which are presented as mean ± standard deviation. Statistical significance was determined by Student’s *t* test based on *p* < 0.01 (*) and *p* < 0.001 (**).

A positive effect of compounds **I** and **IX** on the root system of plants was previously established [3]. Our experiments showed that the tested compounds **II**, **III**, and **V**–**VII** had a positive effect on the architecture of roots, promoting the formation of main and lateral roots (the total number of roots was six). In contrast to the shoots treated with **I**, those treated with **IV**, **VIII**, **IM**, and **KIN** and the control plants (Figure 4) had mainly four roots. It should be noted that shoots treated with compounds **II** and **VI** had lateral roots with root hairs. Presumably, this is the consequence of a change in the balance between endogenous cytokinins and auxins under the action of EDU [34].

To assess the water status of a plant at a specific point in time, it is necessary to test the relative water content (RWC) of the leaves [3,31]. A typical RWC value is 98% in freshly cut leaves. When the RWC is in the range of 40–70%, the process of gradual leaf withering has begun. A value of RWC between 30 and 40% is the main indicator of when the withering process can be reversed. Severe drying and aging of leaves are observed at RWC values below 20% [3,31]. Higher RWC values are an indicator of drought tolerance [35,36].

**Figure 5:**
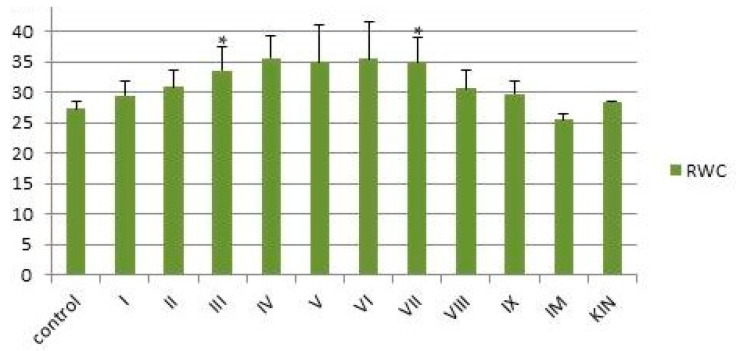
Relative water content (RWC %) of leaves 120 h after the last watering in wheat seeds treated by compounds **I**–**IX**, **IM**, and **KIM** and controls. Online calculator was used to analyze the data, which are presented as mean ± standard deviation. Statistical significance was determined by Student’s *t* test based on *p* < 0.01 (*).

The values of RWC for compounds **I**–**IX** were calculated using Formula (3). The results are shown in Figure 5. The shoots treated by compounds **III**–**VII** 120 h after the last watering had a minimal degree of wilting (RWC 30–35%) compared to the control samples (RWC 25–28%). Thus, compounds **III**–**VIII** increase the drought resistance of plants. These results agree with field tests for compounds **I** and **IX** previously performed on wheat seeds [3].

## 3. Materials and Methods

### 3.1. Chemicals

Arylureas *N*-[2-(2-oxoimidazolidin-1-yl)ethyl]-*N*′-(3-chlorophenyl)urea (**I**), *N*-[2-(2- oxoimidazolidin-1-yl)ethyl]-*N*′-(3,4-dichlorophenyl)urea (**II**), *N*-[2-(2-oxoimidazolidin-1-yl)ethyl ]-*N*′-(3-chlorophenyl)urea (**III**), and *N*-[2-(2-oxo- imidazolidin-l-yl)ethyl]-*N*′-phenylurea (IX) were synthesized earlier [27,28]. EDU derivatives *N*-[2-(2-oxoimidazolidin-1-yl)ethyl]-*N*′-(4-tolyl)urea (**IV**), *N*-[2-(2-oxoimidazolidin-1-yl)ethyl]-*N*′-(2,4-dimethylphenyl)urea (**V**), *N*-[2-(2-oxoimidazolidin-1-yl)ethyl]-*N*′-(2,6-dimethylphenyl)urea (**VI**), *N*-[2-(2-oxoimidazolidin-1-yl)ethyl]-*N*′-(4-methoxyphenyl)urea (**VII**), and *N*-[2-(2-oxoimidazolidin-1-yl)ethyl]-*N*′-(2-ethylphenyl)urea (**VIII**) were obtained using the known method (according to Figure 2) from 2-imidazolidinone with an aminoethyl substituent by interaction with various arylisocyanates in the presence of triethylamine in anhydrous acetonitrile (see Supplementary Materials, Figures S1–S10). The structures all of the compounds were confirmed by ^1^H NMR spectroscopy and mass spectrometry. The purity of the compounds was between 95 and 99%, as established by HPLC-MS. Kinetin (KIN) (CAS: 525-79-1), ethylenediurea (EDU) (CAS: 54924-46-8) and N-2-hydroxyethyl-2-oxo-imidazolidine (IM) (CAS: 3699-54-5) were purchased from Sigma-Aldrich (St. Louis, MO, USA).

Wheat seeds (*Triticum aestivum* L.) of the “AGATA^®^” No. 8853479 [37] variety, crop 2020, provided by LLC “Zhito,” Russia 54.60° S.w., 39.80° V.D. were used.

### 3.2. Instruments

^1^H NMR spectra were recorded using a Bruker DRX-400 NMR spectrometer at 400.13 MHz. DMSO-d6 was used as a solvent and TMS as an internal standard. Chemical shift values were measured with an accuracy of 0.01 ppm; coupling constants are given in Hertz. HPLC-MS was recorded on an x Series II ICP-MS inductively coupled plasma mass spectrometer (Thermo Scientific Inc., Waltham, MA, USA). Melting points were determined using a Stuart SMP20 instrument (Cole-Palmer, Stone, Staffordshire, UK). Reaction mixture composition qualitative analysis was performed by thin-layer chromatography on silica gel (0.015–0.040 mm) aluminum-backed TLC plates with an F254 fluorescent indicator (20 × 20 cm) (Merck Millipore, Darmstadt, Germany). “Kieselgel 60” (0.015–0.040 mm, Merck Millipore, Darmstadt, Germany) silica gel was used for chromatographic separation on a preparative scale. 

### 3.3. Laboratory Experiment

Four identical chambers with UFO-79–01-00 phyto-LED lighting with a wavelength of Red 615/Blu 457 nm and an intensity of at least 250 lux were simultaneously used to conduct four series of experiments. The experiment was carried out for 7 days with illumination day/night 12/12 at a relative humidity of 24% and temperature of 20 °C.

The technique described in [3,31] was used to spray the product on the 50 dry sterilized wheat seeds placed on filter paper in 75 *×* 85 (mm) Petri dishes. All tested compounds are highly soluble in water. Aqueous solutions with concentrations of 4 × 10^−5^ M were prepared for all test compounds according to a known technique [31]. Wheat seeds were sprayed with 0.34 ± 0.02 mL of the test compound solutions.

The germination potential of wheat seeds was determined 24 h after the start of the experiment according to Formula (1):

**Germination potential (%) = [Number of germination seeds 1d*/*Number of total seed] × 100** (1)

Seed germination was calculated by Formula (2) [30] after the end of the experiment:

**Germination (%) = [Number of germination seeds 7d*/*Number of total seed] × 100** (2)

Root lengths, shoot heights, and relative water content (RWC) were determined from fifteen randomly selected shoots from Petri dishes. The last watering was 96 h after the start of the experiment. RWC was determined within three days after the end of the experiment according to Formula (3):

**RWC % = [(TW (FW − DW)/−DW)] × 100** (3)

where FW = fresh weight; TW = turgid weight; DW = dry weight.

### 3.4. Statistic

The relationships between the studied indicators were determined using the statistical software package “Statistica 12.1” (Stat-Soft Inc., Tulsa, OK, USA). Data are presented as mean ± standard deviation. Statistical significance was determined by Student’s *t* test at significance levels of *p* ˂ 0.05 and *p* ˂ 0.01. Microsoft Excel 2010 and Corel-Photo pain 12 were used to generate graphs to present the results of the statistical analyses.

## 4. Conclusions

New hybrid ethylenediurea (EDU) derivatives, belonging to the class of cytokinin-like compounds, were obtained, and their effect on the growth and development of wheat (*Triticum aestivum* L.) seeds was studied. Compounds **I**–**IX** were available and prepared according to a standard procedure starting from substituted 2-imidazolidinones and the corresponding phenyl isocyanate. Seeds treated by tested compounds **I**–**IX** showed high values of germination potential and seed germination. There was also a significant positive effect on the growth and development of root system architecture and shoot height, as well as high values of plant drought resistance. It can be assumed that the beneficial effect on wheat seeds is associated, in particular, with the presence of chlorine atoms in the aromatic ring as an electronegative and hydrophobic substituent, which can lead to increased binding of the urea fragment to cytokinin receptors responsible for the activation of the two-component signaling system. Among the tested compounds, a superior substance, **VIII,** was identified, which showed the best results. These newly studied hybrid derivatives of EDU can be recommended for further study of the possibility of using them in agriculture. In particular, we plan to conduct field tests on wheat seeds under actual drought conditions.

## Figures and Tables

**Table 1:** The results of laboratory tests on the wheat seeds treated with compounds **I**–**IX**. Wheat seeds were treated by compounds **I**–**IX**, **IM**, **KIN**. Germination potential (Gp, **%**), germination (G, %), root length (root, cm), shoot height (shoot, cm), and relative water content (RWC, %) were determined. Seeds in a Petri dish treated only by distilled water were taken as control. **IM** is *N*-2-hydroxyethyl-2-oxo-imidazolidine; **KIN** is N^6^-furfuryladenine. Data are presented as mean ± standard deviation. The statistical significance was determined by Student’s *t* test based on *p* < 0.05 (*) and *p* < 0.01 (**).

Compounds	Gp, %	G, %	Root, cm	Shoot, cm	RWC, %
Control	56.47 ± 4.19	84.21 ± 2.63	7.76 ± 1.93	12.63 ± 3.18	27.29 ± 1.16
**I** 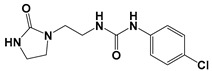	53.28 ± 3.05	92.17 ± 4.34	7.82 ± 1.10	**15.04** ± 2.91 **	29.46 ± 2.37
**II** 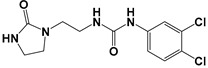	**59.60** ± 1.70 *	**93.51** ± 3.41	8.16 ± 1.70	**15.27** ± 1.82 *	30.84 ± 2.95
**III** 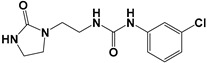	52.41 ± 2.01	**92.54** ± 2.58 *	**8.71** ± 2.08 **	**18.85** ± 3.16 **	33.58 ± 3.96 *
**IV** 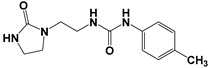	54 ± 3.78	88 ± 2.51	**8.3** ± 1.29 *	**5.4** ± 1.58 *	35.58 ± 3.81
**V** 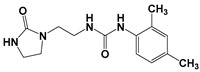	51.33 ± 2.08	85.62 ± 2.36	**8.27** ± 1.82 *	**14.42** ± 1.20 *	35.02 ± 6.08
**VI** 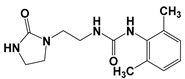	52.05 ± 5.5	88.31 ± 5.91	**9.62** ± 1.29 **	13.50 ± 1.83	35.52 ± 6.40
**VII** 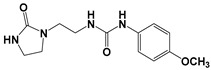	65.39 ± 2.16 *	**94.27** ± 2.82 *	**8.96** ± 1.31 **	**15.47** ± 1.76 *	34.97 ± 4.12 *
**VIII** 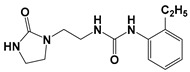	57.45 ± 2.46	90.11 ± 5.56	**9.37** ± 1.82 **	**15.12** ± 1.67 *	30.68 ± 3.08
**IX** 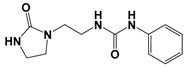	55.61 ± 2.64	86.24 ± 2.16	8.88 ± 1.31	14.34 ± 1.65	29.73 ± 2.05
**IM**	60.05 ± 4.32	86.42 ± 2.0	6.26 ± 1.55	13.50 ± 1.2	25.52 ± 0.89
**KIN**	59.32 ± 6.84 *	88.16 ± 7.79	8.13± 0.55	14.57 ± 2.4 *	28.43 ± 0.04

## Data Availability

Data are contained within the article and Supplementary Materials.

## Supplementary Materials

The supporting information can be downloaded at: https://www.mdpi.com/article/10.3390/ijms25063335/s1.
