# Supplementary material for: New Hybrid Ethylenediurea (EDU) Derivatives and Their Phytoactivity
Source: Int J Mol Sci. 2024 Mar 15;25(6):3335. doi: 10.3390/ijms25063335 (PMC10970543; doi:10.3390/ijms25063335)
Supplement: Supplementary file 1 [file ijms-25-03335-s001.zip › ijms-2863186-supplementary/ijms-2863186-supplementary.pdf]

New Hybrid Ethylenediurea (EDU) Derivatives and Their Phytoactivity

Maxim S. Oshchepkov [1,*], Leonid V. Kovalenko [1], Antonida V. Kalistratova [1], Sergey V. Tkachenko [1], Olga N. Gorunova [2], Nataliya A. Bystrova [2] and Konstantin A. Kochetkov [1,2,*]

[1] Department of Chemistry and Technology of Biomedical Drugs, Mendeleev University of Chemical Technology of Russia, Miusskaya Sq. 9, 125047 Moscow, Russia; kalistratova.a.v@muctr.ru (A.V.K.)
[2] A. N. Nesmeyanov Institute of Organoelement Compounds, Russian Academy of Sciences, 28 Vavilova St., 119991 Moscow, Russia; olg111@yandex.ru (O.N.G.); bystrova17.11@inbox.ru (N.A.B.)
* Correspondence: oshchepkov.m.s@muctr.ru or maxim.os@mail.ru (M.S.O.); const@ineos.ac.ru (K.A.K.)

Content

I. Preparation of substances.
II. Methodology of the experiment
III. Spectral data of compounds (Figures **S1-S10**)

I. Preparation of substances

Arylureas **I-III** were synthesized by the previously [27, 28]. Compounds **IX** is commercially available.

General procedure for the synthesis of compounds I-IX

In a three-neck flask with a thermometer, a dropping funnel and a magnetic stirrer, 31 mmol of 1-(2-aminoethyl)-2-imidazolidinone in 50 ml of dry toluene was placed. The mixture was cooled in an ice bath to a temperature no higher than 5 °C. Then solution of 31 mmol of the relevant phenyl isocyanate in 50 ml of dry toluene was added dropwise with stirring, keeping a temperature not higher than 5 ° C. The reaction mixture was stirred for a day. The precipitate was filtered and recrystallized from acetone.

2-(2-oxoimidazolidin-1-yl)ethyl-N-(*p*-tolil) urea (**IV**). 53% yield. M.p. = 189-190 °C. 1 H NMR (DMSO-d_6, δ, ppm, J, Hz): 2.19 (**s**, 3H, -C<u>H</u>$_3$); 3.07-3.13 (**m**, 2H), 3.15-3.20 (**m**, 2H), 3.20-3.25 (**m**, 2H), 3.31-3.39 (**m**, 2H) (-C<u>H</u>$_2$-); 5.98 (**t**, 1H, -CH$_2$-NH-C(O)-NH-, *J* = 5.4); 6.16 (**s**, 1H, -N<u>H</u>-C(O)-N-); 6.99 (**d**, 2H, *J* = 8.2), 7.23 (**d**, 2H, *J* = 8.4, C<u>H</u>ar); 8.29 (**s**, 1H, -C(O)-N<u>H</u>-Ar). HPLC-MS: $[M + 1]^+$ 263.30; calculated value is 263.31.

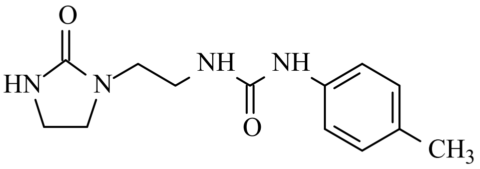

2-(2-oxoimidazolidin-1-yl)ethyl-N-(2,4-dimethylphenyl) urea (**V**) 58% yield. M.p. = 178-181 °C. 1 H NMR (DMSO-d_6, δ, ppm, J, Hz): 2.11 (**s**, 3H, -CH_3); 2.18 (**s**, 3H, -CH_3); 3.09-3.12 (**m**, 2H), 3.16-3.24 (**m**, 4H), 3.34-3.38 (**m**, 2H, -CH_2-); 6.30 (**t**, 1H, -CH_2-NH-C(O)-NH-, *J* = 5.5); 6.16 (**s**, 1H, -NH-C(O)-N-); 6.84-6.87 (**m**, 1H), 6.89-6.90 (**m**, 1H, CHar); 7.51 (**s**, 1H, -C(O)-NH-Ar); 7.51-7.53 (**m**, 1H, CHar). HPLC-MS: $[M + 1]^+$ 277.40; calculated value is 277.34.

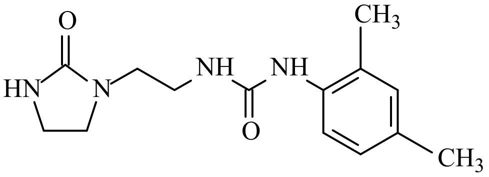

2-(2-oxoimidazolidin-1-yl)ethyl-N-(2,5-dimethylphenyl) urea (**VI**) 62 % yield. M.p. = 212-214°C. ^{1} H NMR (DMSO-d_6, δ, ppm, J, Hz): 2.12 (**s**, 6H, -CH_3); 3.07-3.09 (**m**, 2H), 3.12-3.23 (**m**, 4H), 3.32-3.37 (**m**, 2H, -CH_2-); 6.01 (**t**, 1H, -CH_2-NH-C(O)-NH-, *J* = 5.4); 6.26 (**s**, 1H, -NH-C(O)-N-); 6.97-7.04 (**m**, 3H, CHar); 7.50 (**s**, 1H, -C(O)-NH-Ar). HPLC-MS: $[M + 1]^+$ 277.41; calculated value is 277.34.

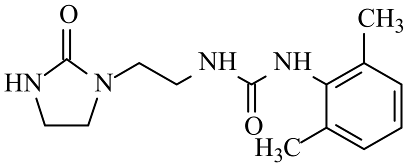

2-(2-oxoimidazolidin-1-yl)ethyl-N-(4-methoxyphenyl) urea (**VII**) 65% yield. M.p. = 155-157 °C. ^{1}H NMR (DMSO-d_6, δ, ppm, J, Hz): 3.11-3.17 (**m**, 2H), 3.17-3.31 (**m**, 4H), 3.35-3.42 (**m**, 2H, -CH_2-); 3.67 (**s**, 3H, -OCH_3); 5.93 (**t**, 1H, -CH_2-NH-C(O)-NH-, *J* = 5.4); 6.16 (**s**, 1H, -NH-C(O)-N-); 6.76-6.87 (**m**, 2H), 7.24-7.32 (**m**, 2H, CHar); 8.20 (**s**, 1H, -C(O)-NH-Ar). HPLC-MS: $[M + 1]^+$ 279.38; calculated value is 279.31.

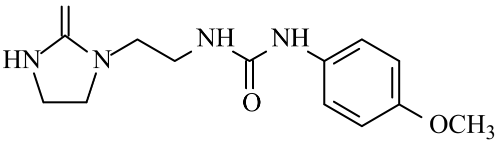

2-(2-oxoimidazolidin-1-yl)ethyl-N-(2-ethylphenyl) urea (**VIII**) 67% yield. M.p. = 148-149°C. 1 H NMR (DMSO-d_6, δ, ppm, J, Hz): 1.10 (**t**, 3H, -CH_3, *J* = 7.5); 2.51 (**q**, 2H, -CH_2-, *J* = 7.9); 3.07-3.12 (**m**, 2H), 3.16-3.23 (**m**, 4H), 3.32-3.38 (**m**, 2H, -CH_2-); 3.67 (**s**, 3H, -OCH_3); 6.30 (**s**, 1H, -NH-C(O)-N-); 6.51 (**t**, 1H, -CH_2-NH-C(O)-NH-, *J* = 5.4); 6.88-6.93 (**m**, 1H), 7.04-7.12 (**m**, 2H,

CHar); 7.66 (**s**, 1H, -C(O)-N$\underline{H}$-Ar); 7.68-7.73 (**m**, 1H, C$\underline{H}$ar) HPLC-MS: [M + 1]$^+$ 277.41; calculated value is 277.34.

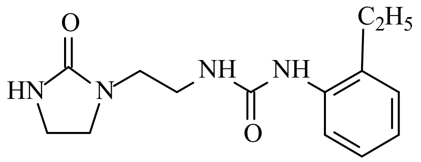

II. Methodology of the experiment.

Before starting work, wheat seeds (*Triticum aestivum* L.) sterilized with 0.2% sodium hydrochloride solution for 10 minutes, washed three times with distilled water, dried at a temperature of 30 ° C for 48 hours. The dried seeds were stored at a temperature of 5 ^{o}C.

Vertical spraying was carried out in an isolated box with disposable screen. After spraying, the screen was removed. The surface of the box was disinfected and created with paper napkins. After spraying, the seeds were covered with filter paper and 10 mL of distilled water was poured. Then the Petri dishes with a lid were moved to the growth chamber. The first 24 h of the experiment were conducted in the dark. The seeds were aired every day. Petri dishes were opened for 25 min, and 5–10 mL of distilled water was added so that the seeds did not dry out. On the third day of the experiment, the lids of the Petri dishes were removed so that the shoots grew.

III. Spectral data of compounds

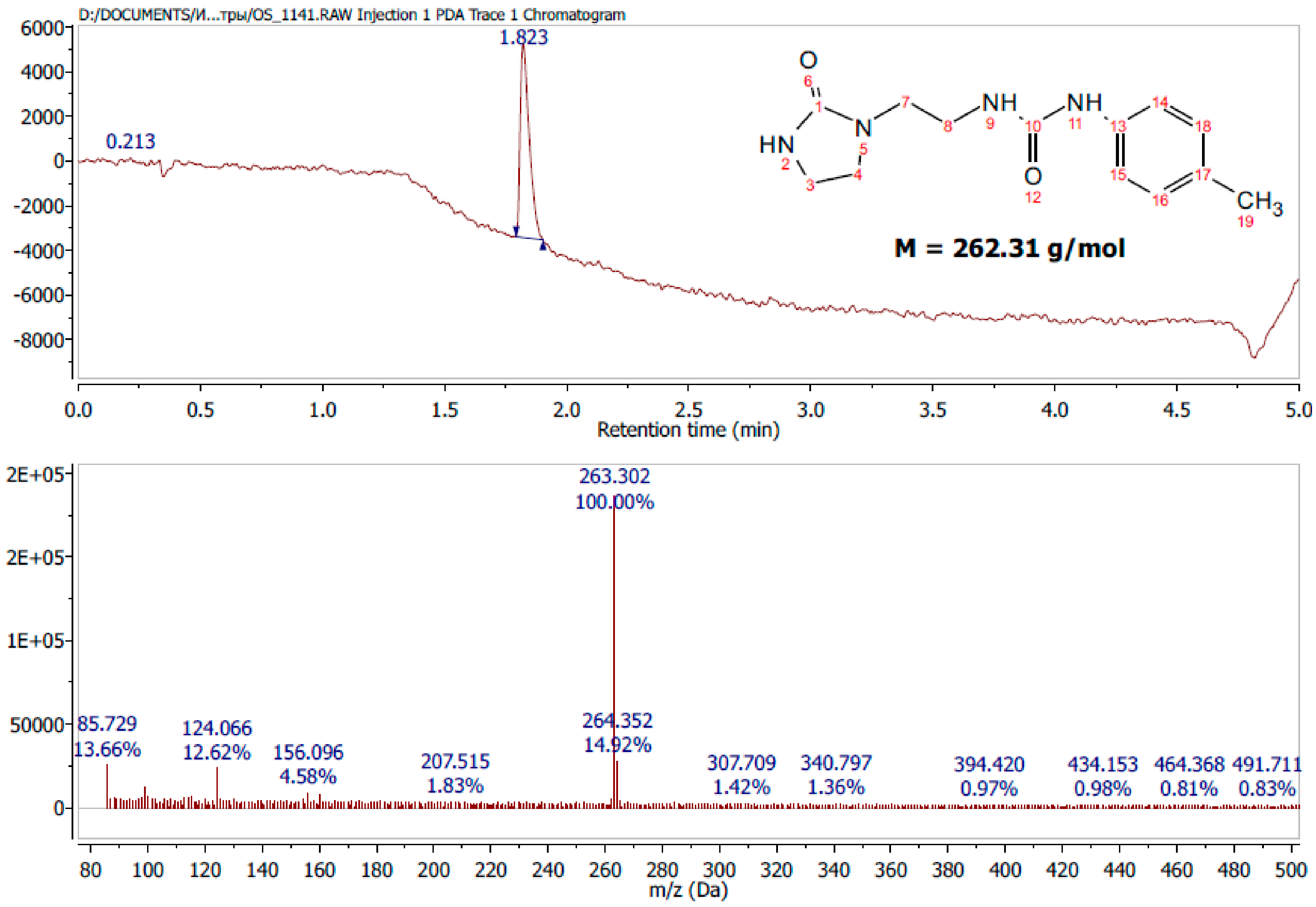

Fig. S1. HPLC-MS spectrum of 2-(2-oxoimidazolidin-1-yl)ethyl-N-(*p*-tolil)urea (**IV**)

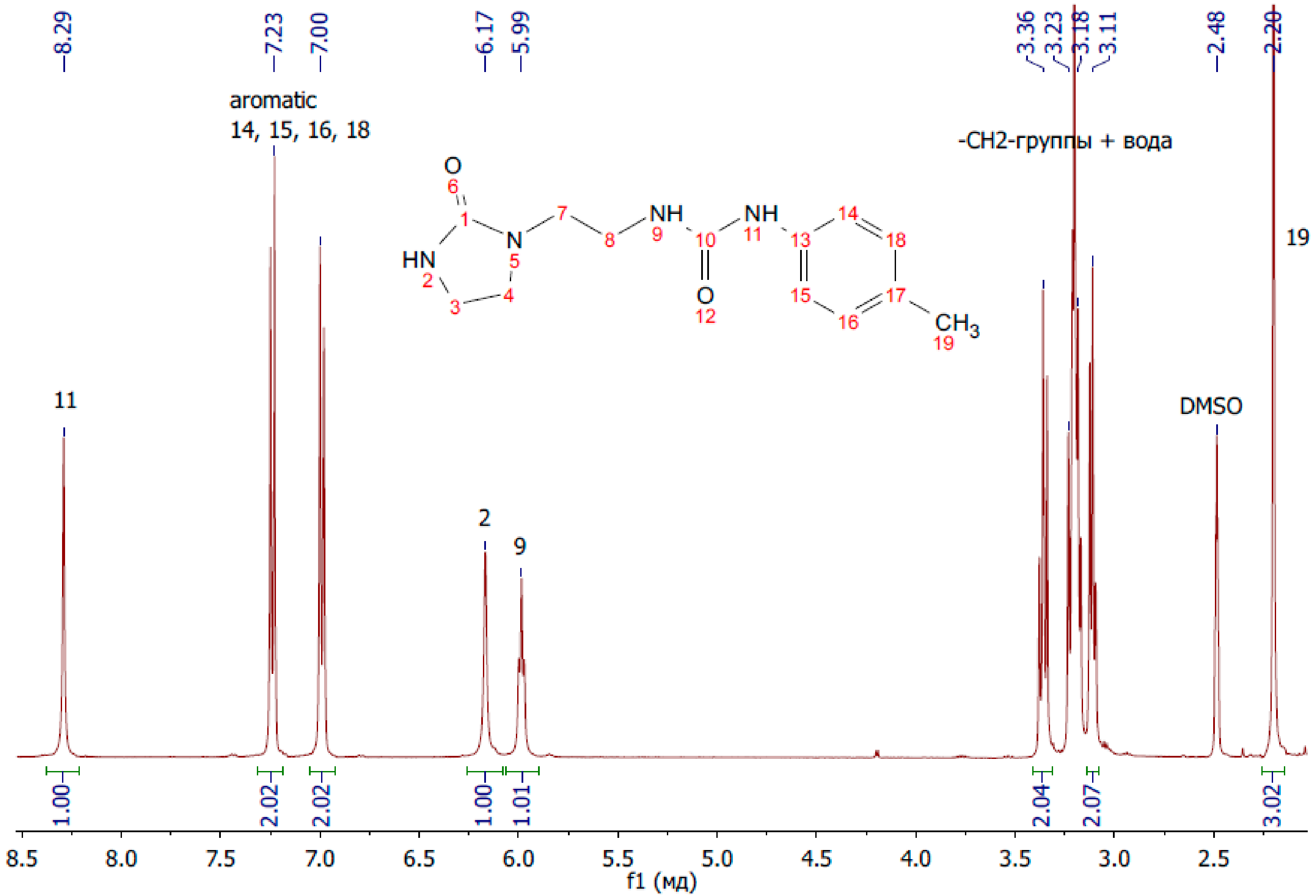

Fig. S2. ^{1}H NMR spectrum of 2-(2-oxoimidazolidin-1-yl)ethyl-N-(*p*-tolil) urea (**IV**)

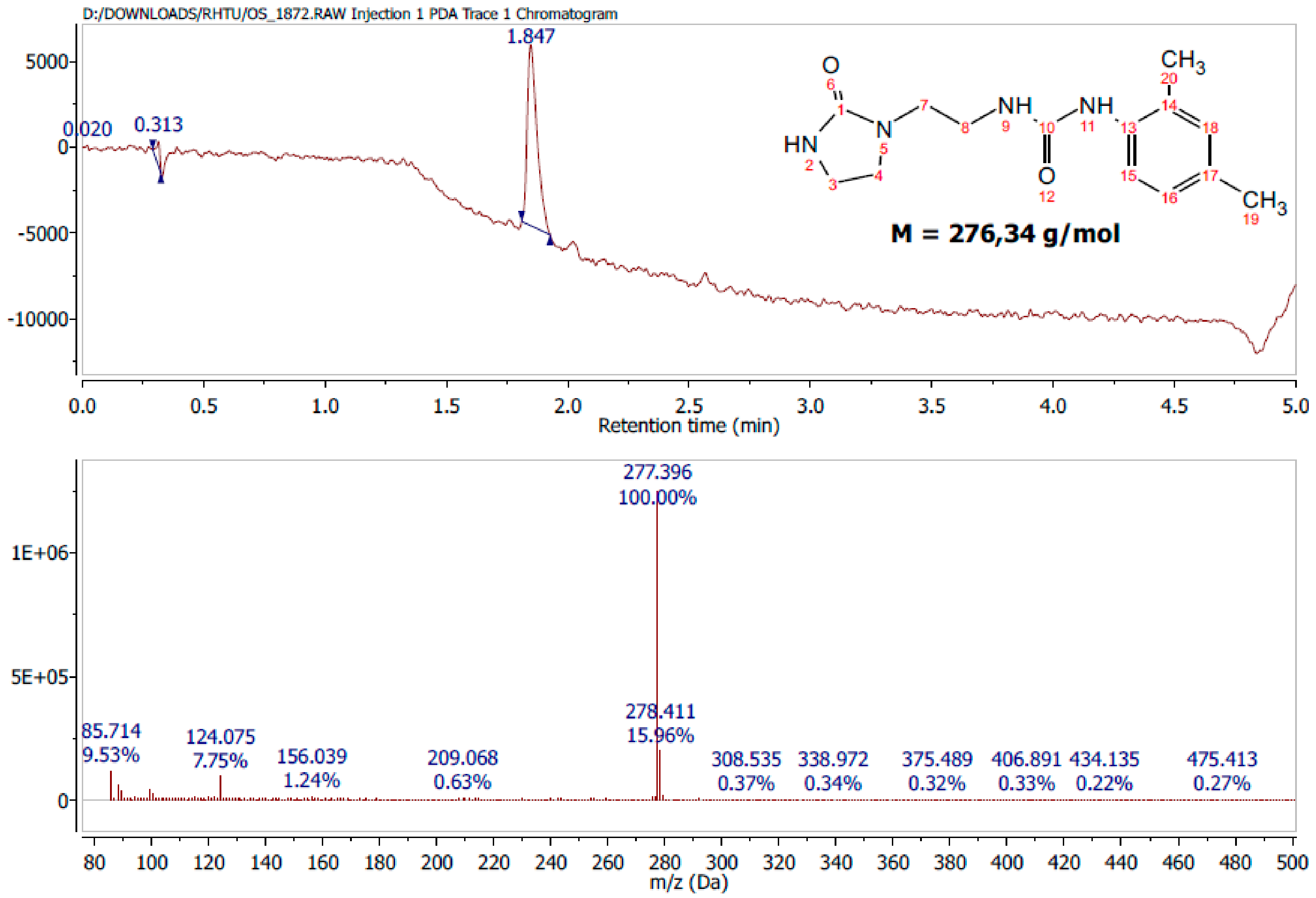

Fig. S3. HPLC-MS spectrum of 2-(2-oxoimidazolidin-1-yl)ethyl-N-(2,4-dimethylphenyl) urea (**V**)

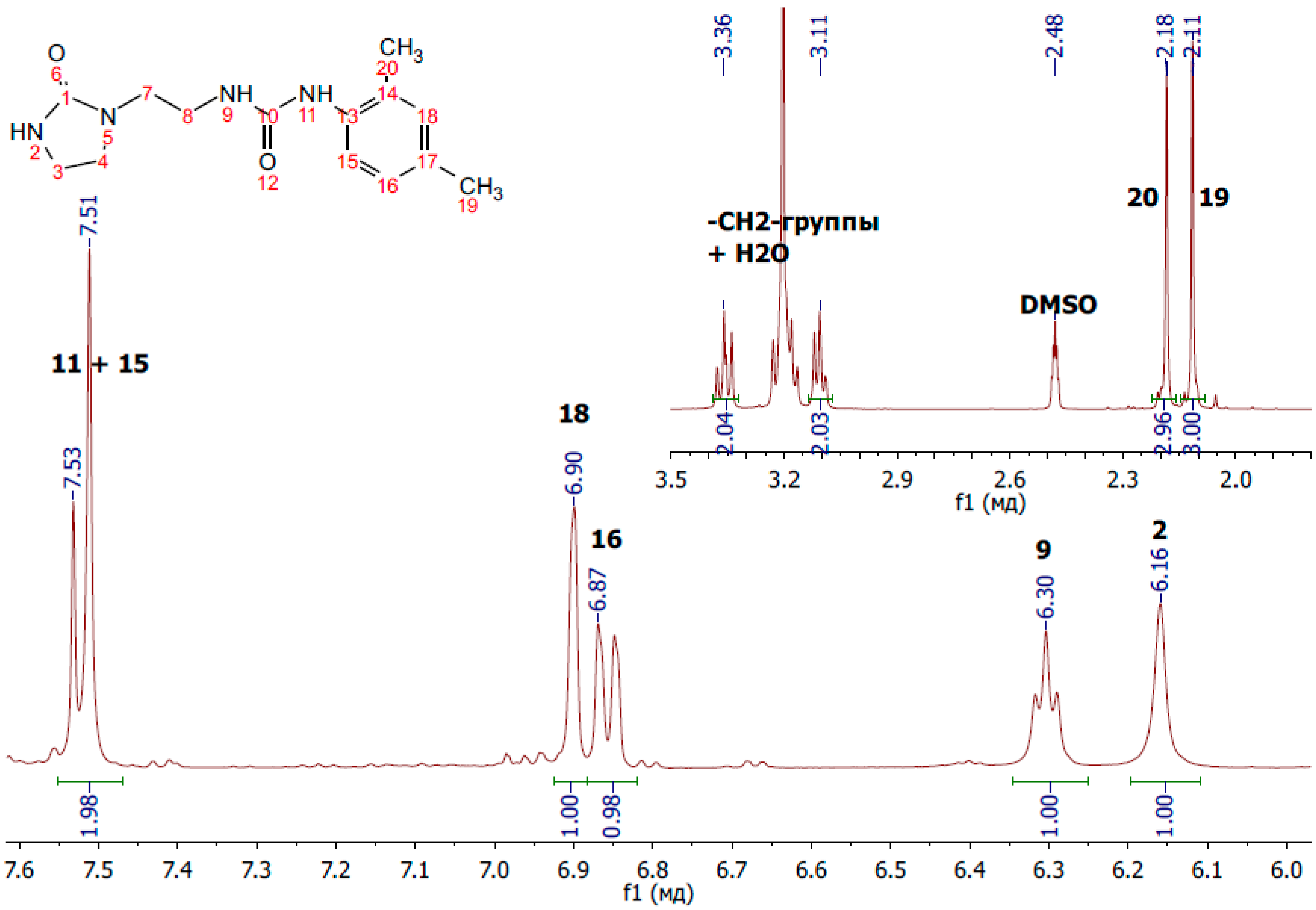

O
6
1
HN
2
3
4
N
5
7
8
NH
9
10
O
12
NH
11
13
14
CH3
20
18
17
CH3
19
15
16
11 + 15
7.53
7.51
18
6.90
16
6.87
9
6.30
2
6.16
1.98
1.00
0.98
1.00
1.00
7.6
7.5
7.4
7.3
7.2
7.1
7.0
6.9
6.8
6.7
6.6
6.5
6.4
6.3
6.2
6.1
6.0
f1 (мд)
3.36
3.11
2.48
2.18
2.11
-CH2-группы
+ H2O
DMSO
20
19
2.04
2.03
2.96
3.00
3.5
3.2
2.9
2.6
2.3
2.0
f1 (мд)

Fig. S4. ^{1}H NMR 2-(2-oxoimidazolidin-1-yl)ethyl-N-(2,4-dimethylphenyl) urea (**V**)

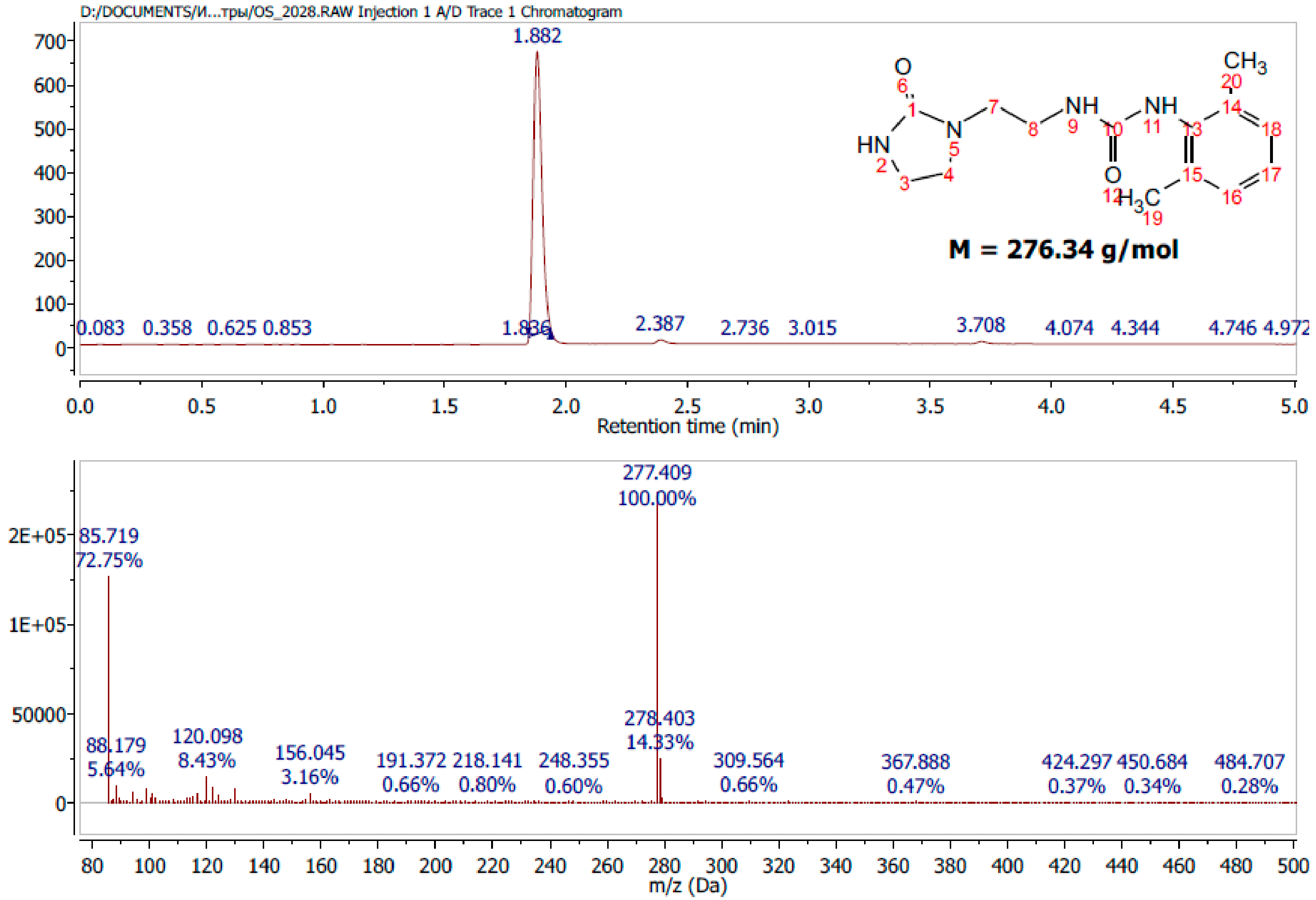

Fig. S5. HPLC-MS spectrum of 2-(2-oxoimidazolidin-1-yl)ethyl-N-(2,5-dimethylphenyl) urea (**VI**)

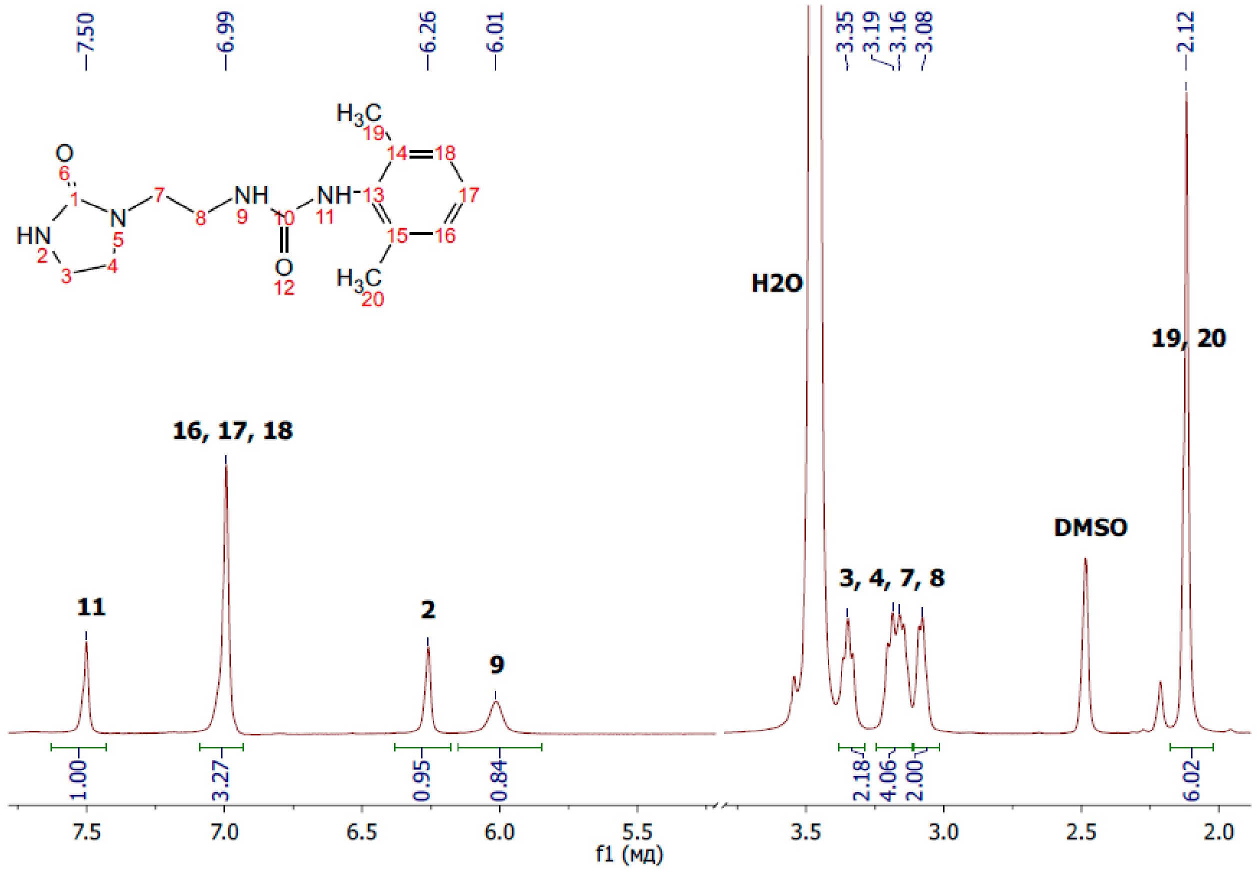

Fig. S6. ^{1}H NMR spectrum of 2-(2-oxoimidazolidin-1-yl)ethyl-N-(2,5-dimethylphenyl) urea (**VI**)

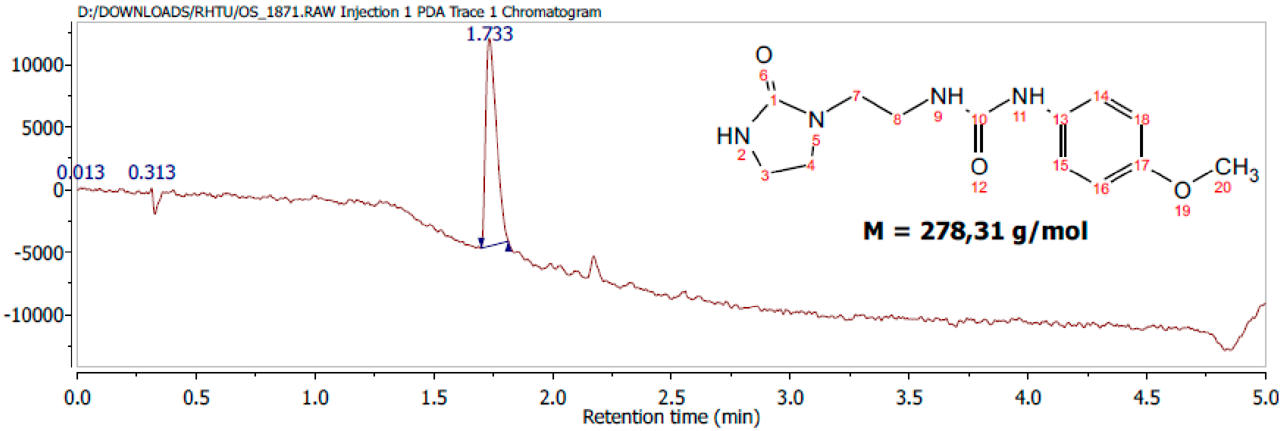
D:/DOWNLOADS/RHTU/OS_1871.RAW Injection 1 PDA Trace 1 Chromatogram
1.733
0.013
0.313
10000
5000
0
-5000
-10000
0.0
0.5
1.0
1.5
2.0
2.5
3.0
3.5
4.0
4.5
5.0
Retention time (min)
O
HN
N
NH
NH
O
O
CH3
M = 278,31 g/mol

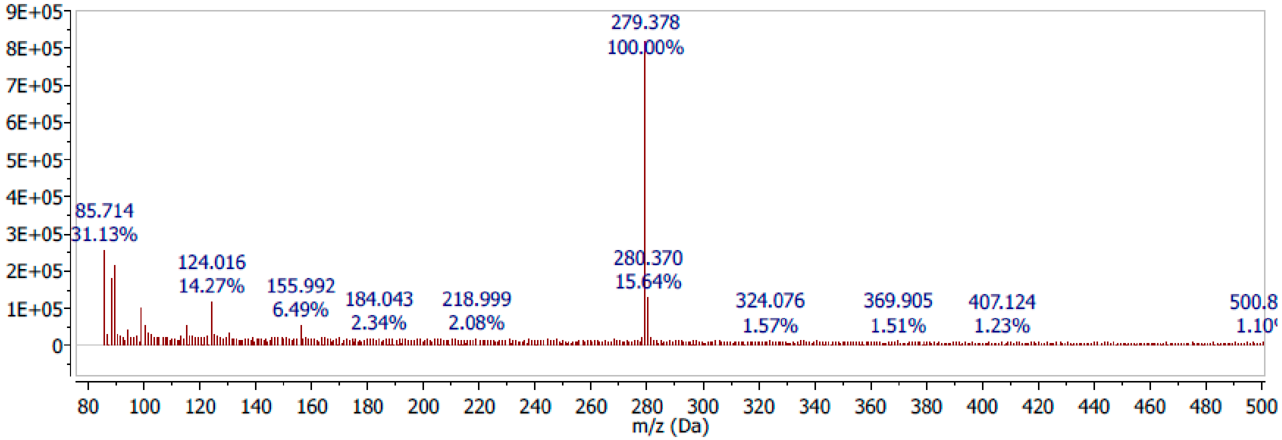
279.378
100.00%
85.714
31.13%
124.016
14.27%
155.992
6.49%
184.043
2.34%
218.999
2.08%
280.370
15.64%
324.076
1.57%
369.905
1.51%
407.124
1.23%
500.8
1.10
9E+05
8E+05
7E+05
6E+05
5E+05
4E+05
3E+05
2E+05
1E+05
0
m/z (Da)

Fig. S7. HPLC-MS spectrum of 2-(2-oxoimidazolidin-1-yl)ethyl-N-(4-methoxyphenyl) urea (**VII**)

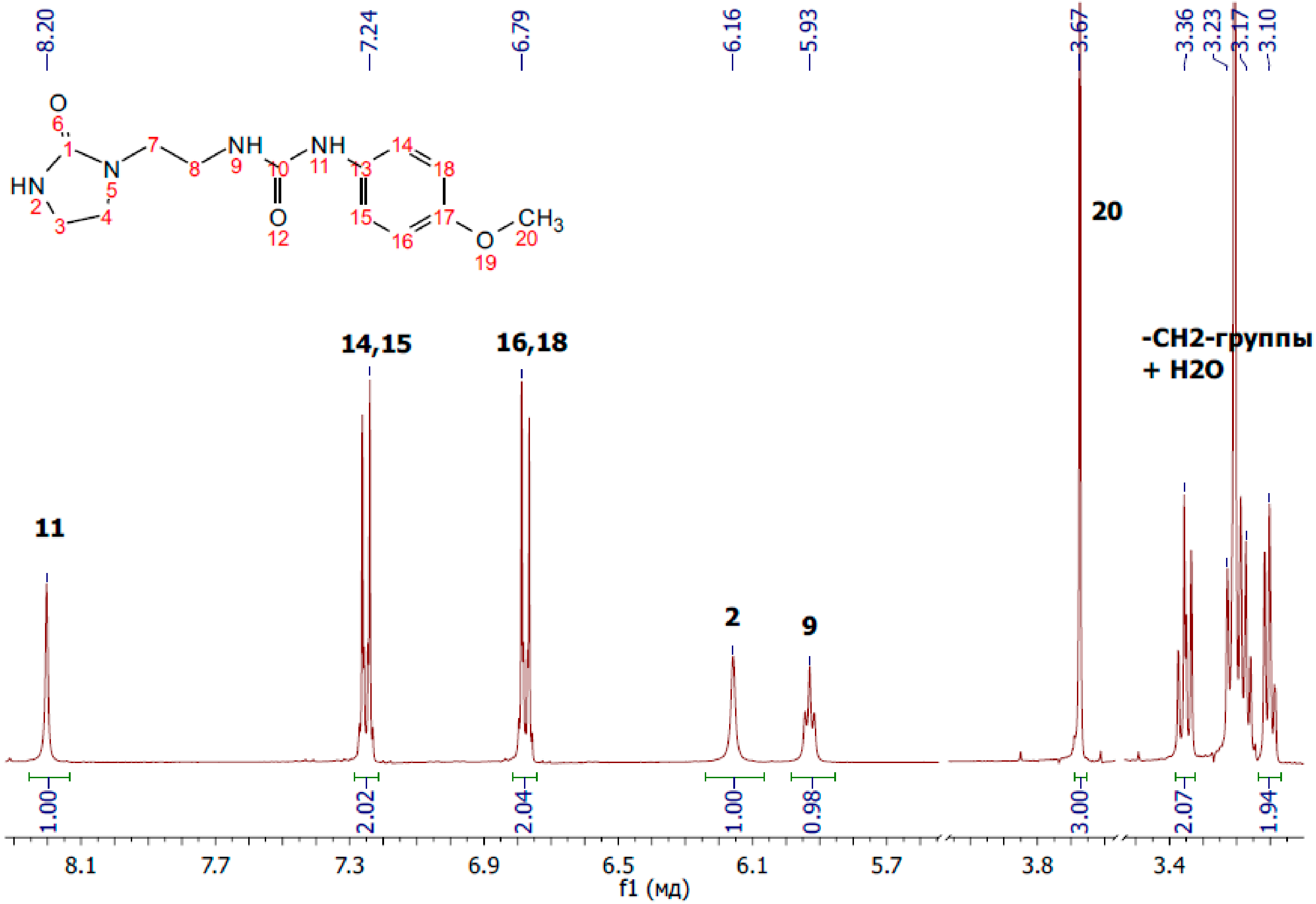

Fig. S8. ^{1}H NMR spectrum of 2-(2-oxoimidazolidin-1-yl)ethyl-N-(4-methoxyphenyl) urea (**VII**)

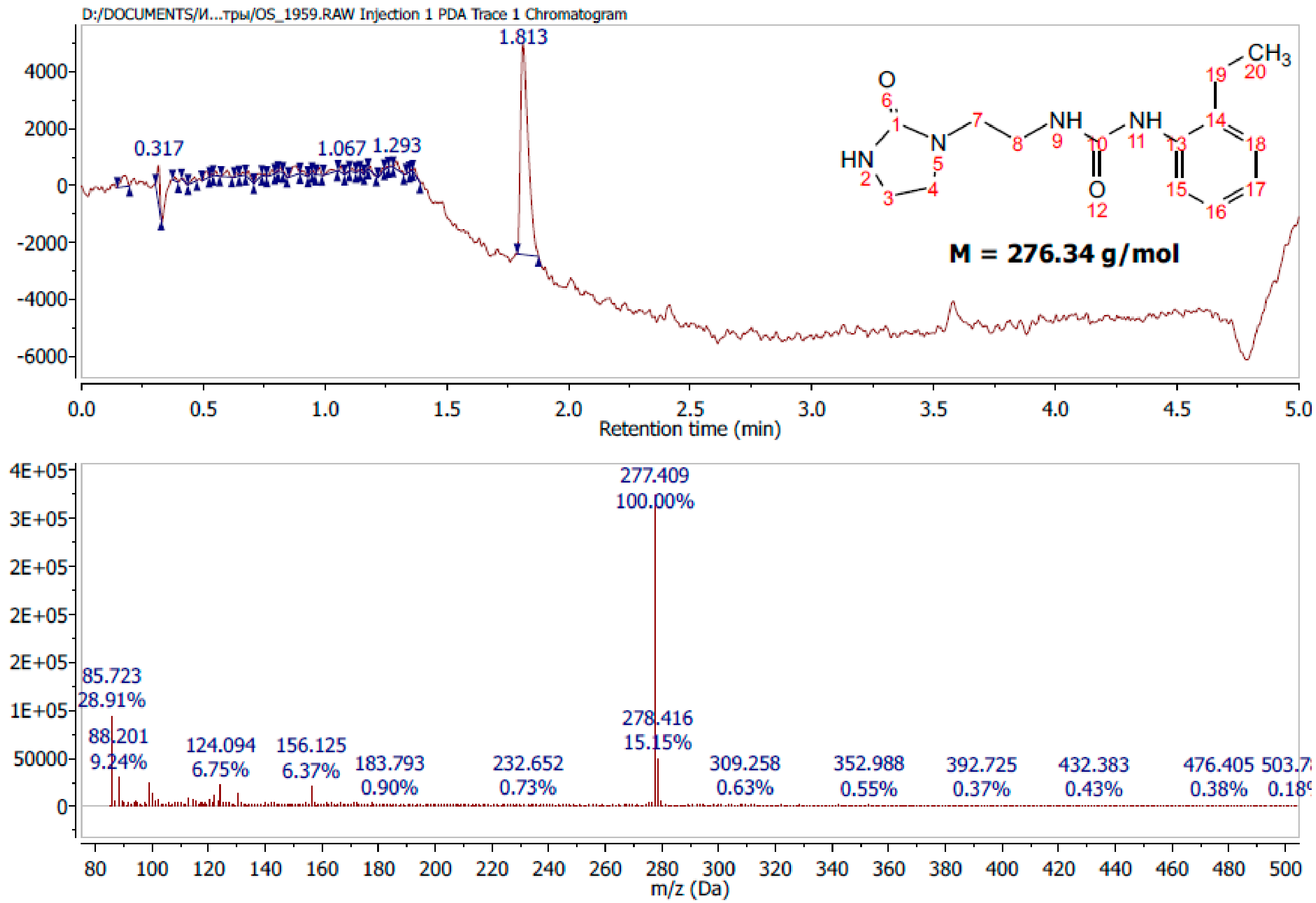

Fig. S9. HPLC-MS spectrum of 2-(2-oxoimidazolidin-1-yl)ethyl-N-(2-ethylphenyl) urea (**VIII**)

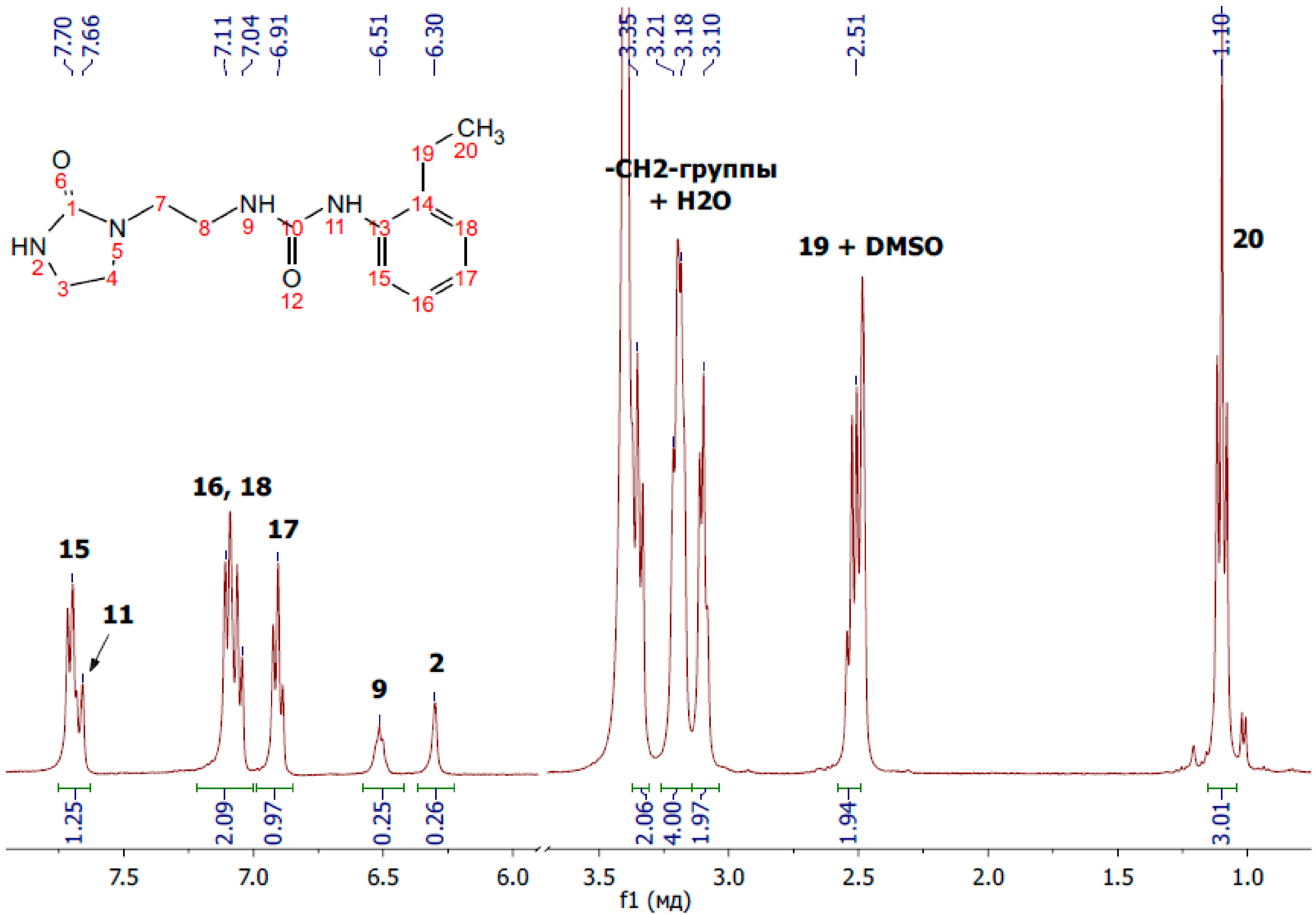

Fig. S10. ^{1}H NMR **2-(2-oxoimidazolidin-1-yl)ethyl-N-(2-ethylphenyl) urea** (**VIII**)
